# Chicago Pathological Society

**Published:** 1889-11

**Authors:** 


### CHICAGO PATHOLOGICAL SOCIETY.

Stated meeting October 1st, Dr. John D. Skeer, president, in the chair.

Dr. Joseph M. Patton read a paper on "Rheumatic Pericarditis."

He said, ætiologically, inflammation of the pericardium stands in much the same position it did ten years since, the pericardium having generally escaped investigation in the hunt after microbes. Recently one observer had succeeded in inducing pericarditis through cultures from an inflamed pericardial surface, in a pericardium previously irritated by turpentine. Bacteria have been found in the products of pericadial inflammation when they were not present in the blood.

With regard to *rheumatic pericarditis* we know as much as we do about rheumatism. According to Bamberger about thirty per cent. of the cases of pericarditis occur in connection with acute articular rheumatism. Clinically we are not able to tell which cases are liable to this complication, further than that it more often occurs in those where several joints have been successively attacked, and the rheumatic manifestations shifting in character. Da Costa states that *endo* and pericarditis generally attend each other, and that pericarditis without endocarditis is more frequently met with than the contrary condition. Debilitated persons are more likely to have pericarditis without endocarditis complicating rheumatism than robust persons ; in the latter the cardiac trouble is generally endocarditis.

*The subjective symptoms* are not at all characteristic occurring in the course of acute articular rheumatism. There is nothing to particularly emphasize the onset. As a rule there will be no rigor, little or no additional rise in temperature ; the pulse may be accelerated or retarded ; pain to the left of the epigastrium and over the cardiac region; palpitation and dyspnœa are the chief signs, yet they may be absent.

*Dyspnœa* may result from passive congestion of the lung, or from pressure from effusion, and from the latter cause may be severe, when there is comparatively little disturbance of cardiac action. Every case of acute rheumatism should be carefully and frequently examined for pericarditis, as it may come on so quietly as to allow of the accumulation of a large effusion before it is discovered.

*The treatment* is to a large extent symptomatic. In recent cases of rheumatic pericarditis it is well to adopt an expectant line of treatment. If the onset is active, with great pain, the local application of a dozen leaches will give great relief. Digitalis, when the heart's action is labored, is very useful. It should be given carefully, and, if myacarditis be present, very sparingly or not at all, as it would be impossible to give enough to be of any use without danger of toxic results. Iron and a nutritive diet are necessary. Iodide of potash with bicarbonate (when the more active rheumatic trouble has already been controlled) is very useful. Rest should be strictly enjoined until exercise can be taken without cardiac excitement. Paracentesis should be performed when pressure from the effusion causes great dyspnœa, or interferes greatly with the action of the heart, The operation may be performed with a trocar; by incising the skin in the fifth intercostal space about an inch from the left edge of the sternum and passing in the trocar; the stylet should be sheathed as soon as the pericardium is reached; the trocar being passed obliquely, the canula can then be pushed further in if necessary. The aspirator is the preferable instrument for this operation; a needle about one millimetre in diameter should be used; or if the diagnosis or quantity and location of fluid are not clear, one-half as large as puncture of the heart would then cause no trouble. The reversible aspirator with siphon attachment is preferred by some, as the fluid may be flaky and obstruct the needle, in which instance the action may be reversed and the needle cleared without removal. The patient may be in the reeling or recumbent position.

Mr. Henry Layman, in opening the discussion, said: Dr. Patton has gone over the ground so thoroughly that I have but very little to add. It might be of some interest, however, to relate a case which recently came under my observation, which illustrates the connection between this disease and a rheumatic diathesis.

A patient about 25 years of age had been suffering with articular rheumatism in different parts of the body for a considerable time. The case at first seemed to be uncontrollable by treatment. The disease would attack one joint, and then another. A short time before the case came under my observation the patient had developed mitral regurgitation. He gradually improved from this, gaining flesh and strength steadily, but finally began to complain of a severe pain in the pericardium, having all the symptoms of pericarditis which the doctor has described.

It is very interesting to watch the evolution of the disease, commencing with a double rasping murmur, developing in the area described by the essayist, the sounds diminishing in their intensity as the swelling increased, and finally disappearing—reappearing as the swelling subsided, becoming extinguished as the person approached convalescence, the old endocardial sounds remaining as before. The patient to which I refer was so badly prostrated for a time and so ill that I had very little hopes of his life; but he made a good recovery, though badly crippled from pericardial inflammation and pericardial adhesions, and will doubtless develop hypertrophy of the heart in the course of time.

The treatment was essentially that outlined by Dr. Patton—iodide of potassium, and digitalis when indicated. At one time he was given tincture of veratrum veride. The pulse was quite rapid; the drugs were given cautiously, and the patient watched constantly to avoid any depression.

Dr. I. N. Danforth: I believed the essayist failed to allude to the examination of the urine. In my experience, I do not remember of ever having seen a case of acute endo or pericarditis of rheumatic origin without there has been some degree of albuminuria sometimes with casts, often with blood casts, or at least a few stray blood globules; consequently I think that that factor ought to have been incorporated in the paper to make it complete.

Then there is another point which struck me very forcibly, and that is the use of digitalis to which Dr. Patton alluded. I do not think we need a cardiac tonic as much as we need something to retard the heart's action, and of late years I have been giving aconite or veratrum veride where I formerly administered digitalis. When we know that the heart is already overworked in rheumatic conditions, I am inclined to think that digitalis is likely to do as much harm as it does good, and that it is oftentimes given when it ought not to be. A remedy which quiets or lessens the strength of the pulsations is more appropriate than one which excites or strengthens them. The wonderful efficacy of digitalis in cases where it *is* indicated has a tendency to tempt us all into using it perhaps too generally.

Dr. Wm. Patterson, in closing the discussion, said: I would like to say, that in speaking of digitalis, I simply stated that it was sometimes a good remedy, but should be used with care, and sometimes not at all.

With reference to the therapeutics of a condition of this kind, it is impossible to apply them in a paper such as this, because the remedy that you will give to a patient and the amount of it, depends entirely upon the action of the heart, the condition of the circulation, the arteries and the exact effect you want to produce. There is no condition that I know of in medicine where a man should know beforehand just what he wants a remedy to do, when to give it and when to stop it, as in a case of this kind. For instance, in a case of pericarditis, where there is no endocardial inflammation or lesion, where the heart is healthy, where you have nervous symptoms characterized by palpitation, I would give *veratrum veride* where the heart is irritable, worked too hard, not because it has any extra work to do, but simply because it is excitable. The only instance in which I would give digitalis would be where there was an actual failure of the heart muscle to do its work. If you have a heart that has been affected with endocardial inflammation, valvular lesion, hypertrophy, etc., you have, supervening on that pericardial inflammation, a tendency towards dilation, because the heart muscle is not of sufficient integrity. You have slight œdema of the lungs through passive congestion. You are getting into trouble simply because you have not sufficient pumping power, and by giving a cardiac tonic you will obtain relief. Digitalis will lower the number of heart beats, increase the strength, and the tension of the blood vessels will increase and dyspnœa will be relieved. *Caffein* will do the same thing; convallaria maialis will sometimes do likewise. Any of the remedies mentioned in such cases are efficient.

As regards the production of albuminuria, I can recall several cases where it was present, but they were all cases of chronic rheumatism—that is, there had been rheumatic manifestations in different ways for several years, and there was rheumatic endocarditis. Whether the albuminuria was due to kidney complications I am not able to say. I do not think I can recall a case of primary pericarditis, rheumatic or otherwise, where albuminuria was present, but if I should find such a condition, I should expect it to be where there was failure of the heart's action, and due to congestion of the kidneys. In cases of chronic rheumatism, the point would be whether it was due to failure in the circulation or actual involvement of the kidney in rheumatic trouble.

